# Validation of a measuring technique with computed tomography for cement penetration into trabecular bone underneath the tibial tray in total knee arthroplasty on a cadaver model

**DOI:** 10.1186/1471-2342-14-29

**Published:** 2014-08-27

**Authors:** Hennie Verburg, Laurens C van de Ridder, Vincent WJ Verhoeven, Peter Pilot

**Affiliations:** 1 Department of Orthopaedics, Reinier de Graaf Groep, Reinier de Graafweg 3, 2625 AD Delft, The Netherlands; 2 Department of Nuclear Medicine, Reinier de Graaf Groep, Reinier de Graafweg 3, 2625 AD Delft, The Netherlands

**Keywords:** Computed tomography, CT scan, Cement penetration, Total knee arthroplasty, TKA

## Abstract

**Background:**

In total knee arthroplasty (TKA), cement penetration between 3 and 5 mm beneath the tibial tray is required to prevent loosening of the tibia component. The objective of this study was to develop and validate a reliable *in vivo* measuring technique using CT imaging to assess cement distribution and penetration depth in the total area underneath a tibia prosthesis.

**Methods:**

We defined the radiodensity ranges for trabecular tibia bone, polymethylmethacrylate (PMMA) cement and cement-penetrated trabecular bone and measured the percentages of cement penetration at various depths after cementing two tibia prostheses onto redundant femoral heads. One prosthesis was subsequently removed to examine the influence of the metal tibia prostheses on the quality of the CT images. The percentages of cement penetration in the CT slices were compared with percentages measured with photographs of the corresponding transversal slices.

**Results:**

Trabecular bone and cement-penetrated trabecular bone had no overlap in quantitative scale of radio-density. There was no significant difference in mean HU values when measuring with or without the tibia prosthesis. The percentages of measured cement-penetrated trabecular bone in the CT slices of the specimen were within the range of percentages that could be expected based on the measurements with the photographs (p = 0.04).

**Conclusions:**

CT scan images provide valid results in measuring the penetration and distribution of cement into trabecular bone underneath the tibia component of a TKA. Since the proposed method does not turn metal elements into artefacts, it enables clinicians to assess the width and density of the cement mantle *in vivo* and to compare the results of different cementing methods in TKA.

## Background

In total knee arthroplasty (TKA), up to ninety percent of the orthopaedic surgeons use cement to fixate the tibia component [1]. Despite this fixation, one of the main reasons for late failure of TKA is loosening of the tibia component [2]. This loosening is believed to be caused by micro motion at the cement-bone interface. The degree of fixation at the cement-bone interface, and hence the prevention of micro motion, is thought to depend on the penetration depth of cement into the trabecular bone underneath the tibial tray [3,4]. In a post-mortem retrieval analysis of fixation strength of cemented tibial trays, Gebert de Uhlenbrock et al. concluded that fixation is improved by greater cement penetration [5]. Other studies on tibia cement penetration in TKA have shown that cement penetration between 3 and 5 mm beneath the tibial tray appears to be the optimal depth [6,7]. Cement penetration to a depth of less than 3 mm results in a weak cement-bone interface, which predisposes for micro motion. However, penetration deeper than 5 mm can lead to heat-induced necrosis of bone and does not increase the strength of the interface [8,9].

While these studies have demonstrated the importance of optimal cement penetration, measuring the cement mantle surrounding implants *in vivo* remains a challenge. In current clinical practice, cement penetration is measured *in vivo* by means of conventional radiographs [8]. However, the downside of conventional radiographs is that they merely provide a two dimensional view of the cement-bone interface. Moreover, it is difficult to shoot the X-ray beam exactly parallel to the prosthesis. Consequently, the measurement of the penetration depth will not always take place directly under the prosthesis, which is the place where an optimal interdigitation between bone and cement is required for fixation of the tibial tray when using the most common cementing technique, i.e. surface cementing.

Various authors used Computed Tomography (CT) imaging to examine the cement mantle around implants [5,10-14]. Goodheart et al. [11] and Mann et al. [13] used micro-CT image processing, a technique which cannot be used *in vivo*. CT images, on the other hand, can be used *in vivo*. The disadvantage of using CT images used to be that the metal prosthesis produced artefacts in the images, which made a good interpretation difficult. In 2009, Lui et al. [12] attempted to develop an algorithm for reducing metal artifacts in CT images of implanted metal orthopaedic devices. Since then, with the recent technical improvements of the CT equipment and software, this problem might have been solved, and CT images might have become a valid measuring technique for tibia cement penetration in TKA. We hypothesized that it might be possible to examine a cross section (i.e. a transversal CT slice) of the cancellous bone, and to assess the cement distribution on that cross section by calculating the percentage of the cement-penetrated cancellous bone versus the non-penetrated cancellous bone. By examining cross sections at consecutive depths, we might measure the width of the cement mantle as well as the density of the cement penetration at the required depth*.*

So far no measuring technique has been validated for measuring tibia cement penetration in TKA *in vivo* by means of CT imaging. Hence, the objective of this study was to develop and validate an *in vivo* measuring technique which provides a truthful indication of the percentage distributed and penetrated cement in the total scanned surface underneath a tibia prosthesis. We assessed the accuracy of using CT scan images to measure cement distribution, i.e. the amount of cement on a cross section of the bone, and cement penetration, i.e. the amount of cement in the depth of the bone just underneath the tibial tray, and we evaluated the influence of metal elements on the depiction of cement on the CT reconstruction.

## Methods

To be able to discriminate between cement-penetrated cancellous bone and non-penetrated cancellous bone, we first defined the radiodensity (Hounsfield units, HU) ranges for trabecular tibia bone, polymethylmethacrylate (PMMA) cement and cement-penetrated trabecular bone. The calibration of the HU levels was checked by scanning a phantom with water, teflon and nylon compartments. The scan of this phantom was analyzed to determine uniformity between the measured HU levels of water, teflon and nylon. If a parameter exceeded its criteria, corrective measures were taken. Next, we measured cement penetration at various depths after cementing two tibia prostheses, one of which was subsequently removed again to examine the influence of the metal tibia prostheses on the quality of the CT images. This tibia prosthesis had been covered with a silicone layer to enable removal of the prosthesis after the first measurements without damage to the bone-cement interface. The other prostheses remained *in situ* and no silicone was required, therefore we did not use it, as it is not used in the regular procedure of cementing, either. Finally, we controlled the findings on cement penetration by sawing the bones off at the measured depths and taking photographs of the sawed-off surface.

For this study, we used 3 femoral heads which had been removed during total hip arthroplasty. All patients consented to the use of the redundant femoral heads. We prepared these femoral heads on the day of the resection. One femoral head was used to measure the radio-density range of pure PMMA cement and of cement-penetrated trabecular bone, as well as to validate the measured percentage of penetrated cement by comparing it to photographs of the femoral head slices sawed off at the required depth. The other two femoral heads were used as cadaver models for measuring cement penetration depth after cementing a tibia prosthesis into each of them, and one of these two femoral heads was used again for examining the influence of metal tibia prosthesis on the CT images.

The CT slices were analyzed with Osirix® (version 3.2.1 32-bit). OsiriX® is an image processing software dedicated to DICOM images produced by a CT scan and has been specifically designed for navigation and visualization of multimodality and multidimensional images. The photographs of the sawed-off slices were analysed with Adobe Photoshop® CS2 (version 9.0), a graphics editing program that can select an area of an image and sample it for future use.

### HU ranges of trabecular tibia bone

To measure the radio-density range of trabecular tibia bone, we used CT images of 4 patients who had undergone a diagnostic CT scan of the knee. The radio-density range of the area 1 to 6 mm beneath the lowest point of the tibia plateau was defined as the clinically relevant area of trabecular tibia bone. The HU value of this area was measured in transversal and coronal slices.

### HU ranges of PMMA cement

**Figure 1:**
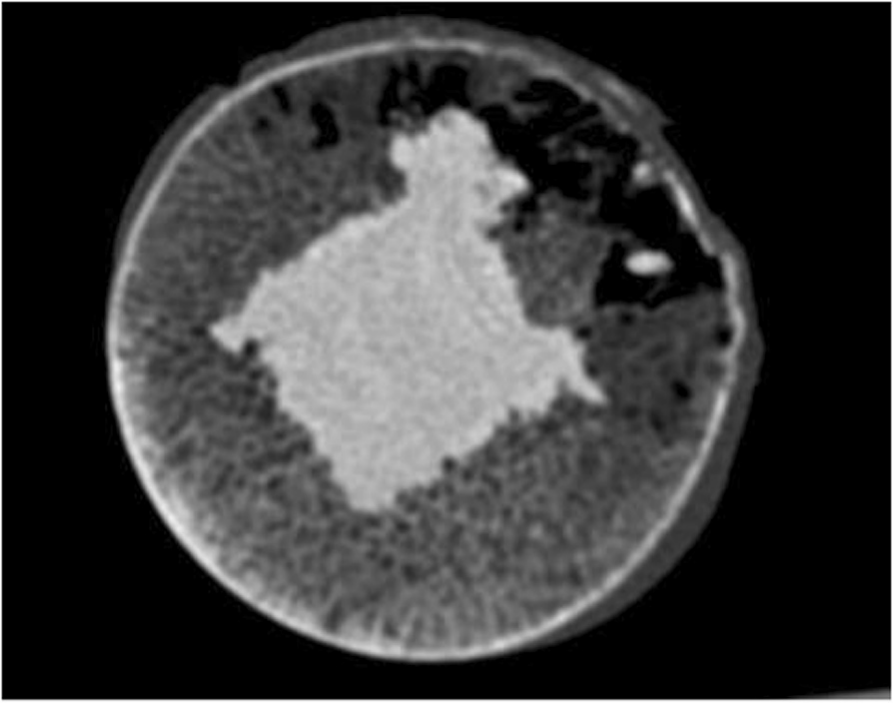
CT scan of the pure cemented cubic centimetre.

To measure the radio-density range of pure cement, we used a slice of one of the femoral heads. From the middle of the cut surface of this slice, 1.0 cubic centimetre of trabecular bone was removed and the hole was filled with cement. Several CT scans were made (Figure 1). Three areas were selected for measurement in transversal slices. In the resulting CT images, radio-density measurements taken in the pure cemented cubic centimetre were defined as the true radio-density range of cement.

### HU ranges of cement-penetrated trabecular bone

To measure the radio-density range of cement-penetrated trabecular bone, another slice of one of the femoral heads was used. The cement was pressurised on top of the cut surface of the slice to achieve good cement penetration into the trabecular bone. Several CT scans were made of the cemented slice. In the resulting CT images, radio-density measurements taken in the transversal slice 3 to 6 millimetres beneath the cut surface were defined as the radio-density range of cement-penetrated trabecular bone.

### Cement penetration at various depths

The tops of two of the femoral heads were sawed off in such a way that the tibial prostheses would fit easily onto the cut surface. The cut surface was prepared for insertion of the smallest NexGen® tibia prostheses (Zimmer®, Warsaw, Indiana, USA), using the NexGen® implantation tools. To cement the tibia prosthesis, the PMMA cement was only applied to the underside of the tibia component and not to the keel. Good compression was achieved by thrusting the tibia prosthesis into the femoral head with a tibial impactor, in line with the way a TKA is usually performed during surgery.

Spiral CT scans were taken of both femoral heads, scanning parallel to the cut surface of the femoral head. The spiral CT scanner (Philips® Gemini GXL 16 slice) produced 0.8 mm slices. The slices were analyzed with Osirix®. Osirix® calculated the mean HU value of the selected areas as well as the standard deviations (SDs) of the mean HU values in the selected areas. These SDs provided an indication of the homogeneity of the scanned material in the selected areas and were identified as the homogeneity values of the selected areas.

By measuring the amount of cement in the cross-sections of every 0.8 mm slice below the underside of the tibial tray, we calculated the penetration depth of the cement in percentages of the trabecular bone surfaces.

### Influence of metal tibia prosthesis on the CT images

**Figure 2:**
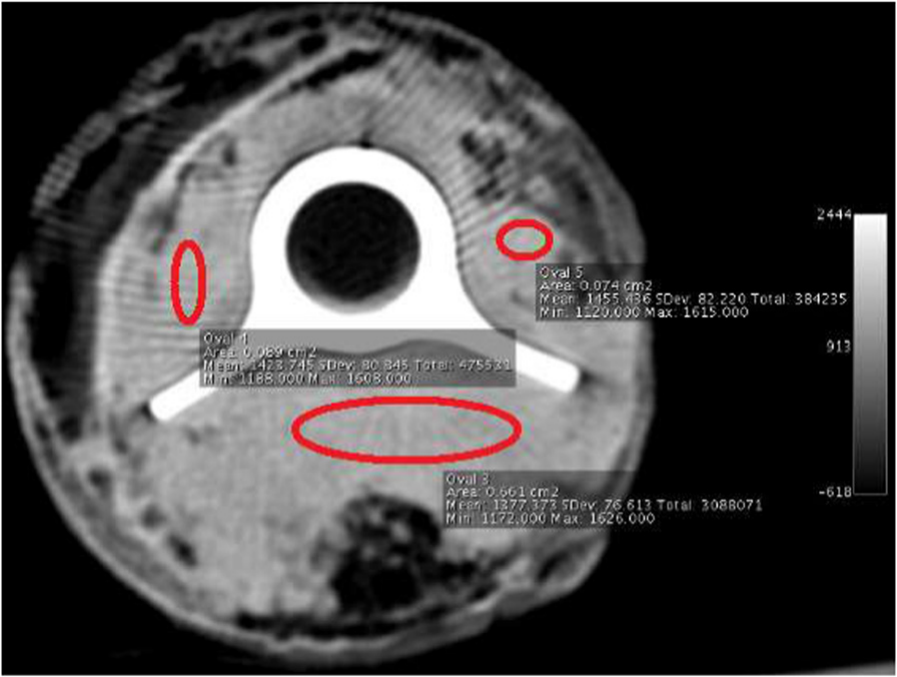
**CT scan of a femoral head 3 mm beneath the cemented tibial tray.** Three areas were selected, two beside the prosthesis and one posterior to the prosthesis.

To assess the influence of metal tibia prosthesis on the CT images, CT scans were made of the femoral head with a tibia prosthesis *in situ* and once again after removal of the tibia prosthesis. Measurements were taken in the transversal slices parallel to the tibial tray. To measure the cement distribution in trabecular bone with the prosthesis *in situ* and without the prosthesis, three areas were selected, two beside and one posterior to the prosthesis (Figure 2).

### Automatic ROI marking

We defined ranges of HU values which enable automatic ROI marking in the CT slices. These ranges were defined to be the mean HU plus and minus twice the mean homogeneity value plus the SD of the mean homogeneity value.

### Validation with photographs

After the radio-density ranges of interest (ROI) were defined, they were used to measure the percentage penetrated cement in the total scanned surface in transversal slices 3 to 6 mm beneath the cut surface. A femur head was cut into halves, both cut surfaces were pressurised with cement and a tantalum bead of 0.5 mm was placed 3 mm beneath the cut surface to facilitate orientation in the CT scans. CT scans were taken of both halves, and the total surface of the transversal slices was measured with Osirix®. Then the radio-density ROI of cement-penetrated trabecular bone was marked to be able to measure the surface of the cement-penetrated trabecular bone area. Next, the radio-density ROI of cement-penetrated trabecular bone was indicated as a percentage of the total scanned surface.

After the CT scans were made, we controlled the findings by means of photographs. A transversal slice was sawed off each half of the femoral head at 3 mm below the cut surfaces, just above the tantalum beads. Both slices were photographed, and the photographs were analysed with Adobe Photoshop® CS2. First the pixels of the total slices were measured, then the colour range of the cement was marked and all pixels in this range were measured. Next, the surface of the cement area was calculated as a percentage of the total slice. The percentages measured in the photographs of the slices were compared with the percentages measured in the corresponding transversal CT slices.

### Statistical analysis

Analyses were performed using SPSS® Statistics for Windows Version 21, Chicago, IL, USA. Differences between the mean percentages of penetrated cement measured in the photographs of the femoral head slices and the percentages measured in the corresponding transversal CT slices were analyzed using the independent t-tests (Student *T*-test). P-values less than 0.05 were considered significant.

## Results

### HU ranges of trabecular tibia bone

In the diagnostic CT scans of four patients, the selected areas in the transversal slices had a mean value of 163 HU (SD 12), with a mean homogeneity value of 152 HU (SD 14). In the coronal slices, the selected areas had a mean value of 162 HU (SD 24), with a mean homogeneity value of 123 HU (SD 16).

### HU ranges of PMMA cement

The mean radio-density value in the selected areas of pure cement was 1328 HU (SD 27), with a mean homogeneity value of 66 HU (SD 12).

### HU ranges of cement-penetrated trabecular bone

The mean radio-density value of cement-penetrated trabecular bone in the selected areas was 1363 HU (SD 30), with a mean homogeneity value of 71 HU (SD 17).

### Cement penetration underneath the tibia prosthesis

The mean value of the selected areas was 1384 HU (SD 53), with a mean homogeneity of 81 HU (SD 30).

### Influence of metal tibia prosthesis on the CT images

Table 1 shows the results of the CT scan measurements of the femoral head with and without the removable tibia prosthesis and of the CT scan measurements of the femoral head with the attached tibia prosthesis. The results indicate that there was no significant difference in mean HU values when measuring with or without the tibia prosthesis.

**Table 1:** HU values of selected areas measured in CT scans with and without prosthesis

	**Femur head with removable prosthesis**		**Femur head without removable prosthesis**		**Femur head with attached prosthesis**	
**CT slice**	**HU value of selected area**	**Homogeneity value**	**HU value of selected area**	**Homogeneity value**	**HU value of selected area**	**Homogeneity value**
1	1377	77	1424	64	1422	129
	1423	81	1350	97	1296	81
	1455	82	1334	76	1402	91
2	1299	122	1395	65	1431	144
	1367	125	1349	89	1433	161
	1262	133	1421	59	1338	53
3	1366	109	1405	39	1534	102
	1372	80	1400	89	1497	104
	1436	116	1386	72	1352	56
4	1304	82	1428	116	1436	143
	1377	93	1478	96	1518	120
	1534	75	1335	97		
5	1413	107	1406	66	1479	159
	1315	120	1340	63	1495	160
	1568	72	1422	69		
Mean (SD)	1391 (84)	98 (21)	1392 (42)	77 (20)	1433 (72)	116 (38)

### Automatic ROI marking

Table 2 shows the HU ranges for automatic ROI marking.

**Table 2:** HU ranges for automatic ROI marking for several materials

**ROI of**	**Range in HU**
Trabecular tibia bone	-192 to 516
Penetrated cement	1062 to 1706
Cortical bone	1386 to 1864
Prosthesis	> 2475

### CT slices compared with photographs of the transversal slices

Table 3 shows the mean percentages of penetrated cement measured in three different photographs of the femoral head slices at 3 mm beneath the cut surface. Table 3 also shows the percentages measured in the corresponding transversal CT slices, defined by means of the tantalum beads, and the slices 0.8 mm beneath the corresponding transversal CT slices. The percentages of measured cement-penetrated trabecular bone in the CT slices of the specimen were within the range of percentages that could be expected based on the measurements with the photographs (p = 0.04).

**Table 3:** Penetrated cement in CT slices and photographs as a percentage of the total transversal slice 3 mm beneath the cut surface

	**Penetrated cement in photographs**	**Penetrated cement in corresponding CT slices**	**Penetrated cement in CT slice 0.8 mm beneath the corresponding slice**
Slice 1	35.4%	39.7%	34.8%
Slice 2	31.3%	34.7%	30.7%
Mean (SD)	33.35% (2.9)	37.2% (3.5)	32.75% (2.9)

## Discussion

The objective of this study was to assess whether CT images can be used to accurately measure the percentage of penetrated cement in the total scanned surface underneath a tibia prosthesis, and to develop and validate a measuring technique that can be used *in vivo* after cementing a total knee arthroplasty. The results showed that trabecular bone and cement-penetrated trabecular bone had no overlap in quantitative scale of radio-density, indicating that this method of measuring cement penetration may be useful in a clinical setting. In addition, there was no significant difference in quantitative radio-density values when measuring with or without the metal prosthesis, indicating that modern CT imaging technology no longer has the disadvantage of turning metal elements into artefacts that confound the interpretation of the images.

Our findings confirm the assumptions of other studies which used CT-scans for femoral implants and defined the Hounsfield Units for (cancellous) bone, PMMA and prosthesis in the same region as we found with our study [10,14].

Our results showed no overlap in the radio-density ROIs of trabecular bone, cement-penetrated trabecular bone and the prostheses. This indicates that when the radio-density ROI of cement-penetrated trabecular bone was registered, there was no trabecular bone or prosthesis in the areas under investigation. Thus it is possible to see the differences between trabecular bone, cement-penetrated trabecular bone or prosthesis in every CT-slice underneath the tibia prosthesis. This enables clinicians to get a good impression of the three-dimensional extent of cement penetration into the trabecular bone after cementing the tibia component of a TKA.

A comparison between the CT slices and the corresponding optical pictures showed different percentages of penetrated cement of the total surfaces of the transversal slices (Table 3). Nevertheless, the percentages of measured cement-penetrated trabecular bone in the CT slices of the specimen were within the range of percentages that could be expected based on the measurements with the photographs (p = 0.04). While the percentages of penetrated cement measured in the corresponding CT slices were higher than the percentages of penetrated cement measured in the photographs, the percentages of penetrated cement in the CT slices 0.8 mm beneath the corresponding CT slices were slightly lower, both 0.6%, than the percentages of penetrated cement measured in the photographs. The difference in percentage of penetrated cement of the total surface can be explained by the fact that the corresponding CT slices did not exactly correspond with the cut surface of the transversal slices. The corresponding CT slices were defined using the tantalum beads as an orientation point, but the CT scans made 0.8 mm slices, implying a possible fault margin of maximally 0.8 mm, which must have resulted in a different cement percentage of the surface. The CT slice of the area slightly above the cut surface of the slice will show a higher percentage of penetrated cement, because the cement in that area met with less resistance and could penetrate to a greater extent.

### Limitations

A limitation of this study was that we used human femoral heads instead of the proximal part of a tibia. The femoral heads were much easier to acquire and we could use them on the same day after the resection during surgery of a total hip arthroplasty. Moreover, there are no big differences between the HU values of cancellous bone in tibia and femoral heads. According to Scheerlinck et al. [14] the HU value of cancellous bone is on average 30 HU (SD 350 HU). Since this is in line with our findings, we consider our results to be relevant for measuring cement penetration in the proximal part of a tibia.

Another limitation was that our findings showed an overlap in the radio-density ROI of cement-penetrated trabecular bone and the radio-density ROI of cortical bone. However, this overlap had only a small influence on the measured percentages of penetrated cement, because only the thin edge on the transversal CT slices consists of cortical bone. Furthermore, all transversal slices had almost the same percentage of cortical bone, and, consequently, the final effect of this overlap in radio-density ROIs was the same in all slices. Therefore we do not expect this to affect the clinical use of the proposed method.

A third limitation is that we compared the cement area to the total scanned surface, without subtracting the metal. However, this did not influence our measurements, since the prosthesis constituted nearly the same percentage of the cross-sectional surface over the measured depth of 3 to 6 mm.

A fourth limitation is that we did not examine the radio-density ROI directly beside the prosthesis. Still, the CT technique we used showed nearly no effect of the metal prosthesis on the surrounding bone/cement. In addition, there was no significant difference in quantitative radio-density values when measuring with or without the metal prosthesis, indicating that modern CT imaging technology no longer has the disadvantage of turning metal elements into artefacts that confound the interpretation of the images.

A final limitation of our study is that with this CT technique, it is impossible to differentiate between bulk cement, like filled bony defects or cysts, and trabecular bone that is interlocked with cement. When cement is pressurized into trabecular bone, the cement fills up the space between the trabecals, which enlarges the density and improves the homogeneity. As a result, the homogeneity value of cement-penetrated trabecular bone is almost the same as the homogeneity value of pure cement. With this result we can make the assumption that the density of cement-penetrated trabecular bone equals the density of pure cement. To differentiate between them, we would have to use micro-CT, which cannot be used *in vivo*. We therefore accepted this drawback as a limitation, since this study aimed to develop and validate a measuring technique that can be used *in vivo* in clinical practice.

### Suggestions for further research

In this study we established that CT imaging can be used to measure the amount of cement at different depths beneath the cutting surface of the femoral head and to calculate the percentage of cement penetrated into the cancellous bone versus the percentage of cancellous bone without penetrated cement. We presented this percentage at a depth of 3 mm beneath the cutting surface, but the same technique can be used to establish the percentage at a depth of 5 mm. Further research might focus on the optimal percentage of cement-penetrated bone that is required at these depths to prevent loosening of the TKA.

## Conclusions

In conclusion, the trabecular cement penetration in TKA can be measured reliably by means of transversal CT slices in combination with the automatically marked radio-density ROI of cement-penetrated trabecular bone and Osirix® computer software. The metal prostheses had no significant effect on the measured radio-density values in the CT images, and there was no overlap between the automatically marked radio-density ROIs of trabecular bone and cement-penetrated trabecular bone. The percentages of cement penetration measured in the CT slices were within the range of percentages that could be expected based on the measurements in the optical pictures. This reproducible technique is, until now, the only way to measure the three-dimensional extent of cement penetration into the trabecular bone after cementing the tibia component of a TKA. Moreover, this technique will make it easier to compare the results of different cementing methods in TKA.

## Abbreviations

TKA: Total knee arthroplasty; CT: Computed tomography; HU: Hounsfield unit; PMMA: Polymethylmetacrylate; SD: Standard deviation; ROI: Range of interest.

## Competing interests

The authors declare that they have no competing interests.

## Authors’ contributions

All authors made substantive intellectual contributions to this research study. HV, LR and PP conceptualized the primary research questions and constructed the study design. LR performed the implantations of the prosthesis and LR and WV assessed the CT scans. HV and LR are responsible for writing this article. All authors read and approved the final manuscript.

## Pre-publication history

The pre-publication history for this paper can be accessed here:

http://www.biomedcentral.com/1471-2342/14/29/prepub
